# Oncologic and perioperative outcomes of laparoscopic versus open radical nephrectomy for the treatment of renal tumor (> 7 cm): a systematic review and pooled analysis of comparative outcomes

**DOI:** 10.1186/s12957-023-02916-y

**Published:** 2023-02-06

**Authors:** Li Wang, Kun-peng Li, Shan Yin, Lin Yang, Ping-yu Zhu

**Affiliations:** 1 Department of Urology, Affiliated Hospital of North Sichuan Medical College, Nanchong, 637000 China; 2 Department of Urology, The Second Hospital of Lanzhou University, Lanzhou, 730030 China

**Keywords:** Laparoscopic radical nephrectomy (LRN), Open radical nephrectomy (ORN), Renal cell carcinoma (RCC), Minimally invasive surgery, Oncological results

## Abstract

**Objective:**

Systematic evaluation of the effectiveness and safety of laparoscopic radical nephrectomy (LRN) for renal tumor (>7 cm).

**Methods:**

The databases PubMed, Scopus, SinoMed, ScienceDirect, and Google Scholar were systematically searched for trials up to November 2022. The pooled results were evaluated by weighted mean difference (WMD), odds ratio (OR), and hazard ratio (HR).

**Results:**

This meta-analysis (18 trials) demonstrated that compared to open radical nephrectomy (ORN), LRN had a longer operative time (OT) (WMD=15.99, 95% CI: 6.74 to 25.24, *p* = 0.0007), lower estimated blood loss (EBL) (WMD = −237.07, 95% CI: −300.02 to −174.12, *p* < 0.00001), lower transfusion rates (OR = 0.37, 95% CI: 0.24 to 0.55, *p* < 0.00001), and shorter length of stay (LOS) (WMD = −2.95, 95% CI: −3.86 to −2.03, *p* < 0.00001). No statistically relevant differences were found in overall survival (OS) (HR = 1.04, 95% CI: 0.81 to 1.35, *p* = 0.76), cancer-specific survival (CSS) (HR = 1.28, 95% CI: 0.97 to 1.68, *p* = 0.08), progression-free survival (PFS) (HR = 1.20, 95% CI 0.97 to 1.48, *p* = 0.1), recurrence-free survival (RFS) (OR = 1.27, 95% CI: 0.89 to 1.81, *p* = 0.56), local recurrence rate (OR = 0.85, 95% CI: 0.42 to 1.71, *p* = 0.65), and intraoperative and postoperative complications.

**Conclusion:**

For patients with renal tumors (> 7 cm), LRN has specific perioperative advantages over ORN (LOS, EBL, and transfusion rates). However, the OT was prolonged in the LRN group. In addition, no differences in complication or oncological outcomes (OS, CSS, PFS, RFS, and local recurrence rate) were reported.

**Trial registration:**

PROSPERO CRD42022367114

**Supplementary Information:**

The online version contains supplementary material available at 10.1186/s12957-023-02916-y.

## Introduction

Renal cell malignant neoplastic disease is the third most common malignancy of the genitourinary system, accounting for approximately 2–3% of cases. Renal cell carcinoma (RCC) incidence and mortality are increasing rapidly due to advances in imaging technology, with some studies indicating an almost exponential trend in North America. Approximately 75,000 cases will be detected in 2021, and over 13,000 people will die from renal cell carcinoma [1, 2].

Current recommendations call for radical nephrectomy for more extensive or locally advanced kidney malignancies that are not amenable to partial nephrectomy (PN). Laparoscopic radical nephrectomy (LRN) is currently the gold standard for stage T1 renal malignant neoplastic illness because it offers equivalent oncological results and perioperative benefits compared to open surgery. The first laparoscopic total nephrectomy was performed in 1991 by Clayman et al. [3]. With the development of surgical techniques, the scope of LRN has expanded to include the removal of localized tumors measuring 7 cm or more. In this case, the LRN would face difficulties narrowing the space and intraoperative vascular and tissue stripping. Despite evidence of the safety and feasibility of LRN for large-volume renal cancer [4, 5], some researchers are cautious [6].

The objective of this systematic review was to provide an evidence-based basis for clinical practice by comparing long-term oncologic outcomes following large-volume renal carcinomas (> 7 cm).

### Evidence acquisition

#### Search strategy

This systematic review and meta-analysis were developed and implemented following the 2020 PRISMA criteria (Preferred Reporting Items for Systematic Reviews and Meta-Analyses) (Table S1). Two reviewers (KP and SY) conducted a thorough search using electronic databases, including PubMed, SCOPUS, SinoMed, ScienceDirect, and Google Scholar. The reference lists of relevant research were also manually searched.

The precision and timeliness of this meta-analysis were verified by searching for study-eligible papers by November 1, 2022, and then again before submission. We searched for the following related terms by intervention and disease: [(laparoscopy OR laparoscopic) AND (laparoscopic radical nephrectomy) AND (open radical nephrectomy) AND (nephrectomy) AND (renal cell carcinoma OR renal tumor OR kidney cancer)]. There were no language restrictions on the search content. The study protocol was registered with PROSPERO (CRD42022367114).

#### Inclusion/exclusion criteria

PICOS (Patient, Intervention, Comparison, Outcome, Study Type) approach is used to establish analysis eligibility and perform follow-up design: (P) adult patients with unilateral renal cell carcinoma greater than 7 cm in size; (I) patients undergoing pure laparoscopic radical nephrectomy (LRN); (C) patients undergoing open radical nephrectomy (ORN); (O) one or more of the following endings exist—oncology-related prognostic outcomes (OS, CSS, PFS, RFS, and local recurrence) and perioperative measures (OT, EBL, LOS, blood transfusion rate, intraoperative and postoperative complication); (S) prospective comparative, retrospective studies, or randomized controlled trials (RCTs).

The following were the exclusion criteria: (1) no relevant data available for the article; (2) no control group in the trial; (3) pure laparoscopic nephrectomy was performed, not hand-assisted or robot-assisted techniques; (4) case reports, expert opinions, reviews, and related animal tests were also excluded.

#### Data extraction and quality assessment

Data were extracted from the baseline characteristics of the study population and related outcome measures using predefined Microsoft Excel. Following the initial screening of titles and abstracts, two researchers (KP and SY) independently read the full text and performed data conversion and extraction. Conflicts were settled by discussion or agreement with a third reviewer (WL). We also contacted the original authors if necessary to obtain complete and usable data. Meanwhile, our staff double-checked references and data for each study to ensure data integrity and feasibility.

The extraction of data included the first author, the publishing year, country, type of research, body mass index (BMI), Proportion of American Society of Anesthesiologists (ASA) score grades ≥ 3, features of renal malignancy (high-grade histological ratio, laterality, inferior vena cava (IVC) thrombus and pathological stage), perioperative-related information (OT, EBL, LOS, blood transfusion rate, and complications), and long-term prognostic outcome of tumors (OS, CSS, PFS, RFS, and local recurrence).

The quality of the enclosed cohort and case-control studies was evaluated using the Newcastle-Ottawa Scale (NOS) [7, 8]. Every NOS item received 1 or 2 points and a score of 5 or less was considered low quality; a score of 6 to 7 was an intermediate grade and a score of 9 or more was considered nearly as good quality.

#### Risk of bias

ROBINS-I (Risk of Bias In Non-randomised Studies of Interventions) is used to evaluate the potential for risk of bias in non-randomized studies when included in a meta-analysis [9].

#### Statistical analysis

The mean and standard deviation (SD) were calculated for continuous numerical variables, but if the raw data provided was only the median and interquartile range (IQR) or the maximum and minimum values of the range. Luo et al.’s [10]. conversion tables were used to transform the data to the normal distribution. For non-normal distribution data, McGrath et al.’s [11] formula was used to provide the corresponding transformation. Oncology outcomes were expressed using hazard ratios HRs and 95% confidence intervals (Cl). Kaplan-Meier curves were used to analyze survival outcomes if they were represented by time-to-event data [12]. The continuous and dichotomous variables involved in the study were expressed as weighted mean differences (WMD) and odds ratio (OR), respectively, and the corresponding 95% Cl.

To assess the degree of heterogeneity between the studies, the *I*^2^ test and chi-square test were used. In statistics, significant heterogeneity was defined as *p*<0.10, suggesting more than moderate heterogeneity (*I*^2^ > 40). *Z* tests were used to determine the statistical significance of the effect sizes of the pooled study and statistical significance was defined as *p* < 0.05. If *I*^2^ > 40%, the random effect models were selected according to the Cochrane review principles. In all other situations, fixed-effect models were applied.

The baseline and tumor characteristics were compared to determine statistical variability between the treatment groups. All statistical analyses were conducted using RevMan 5.3 and Stata 14.0 versions. We investigated the sensitivity of specific studies with substantial heterogeneity using a leave-one-out strategy. Using funnel plots and Egger’s regression tests (*p* > 0.05), publication bias was ostensibly evaluated for results with more than ten studies [13, 14].

## Results

### Baseline characteristics

**Fig. 1:**
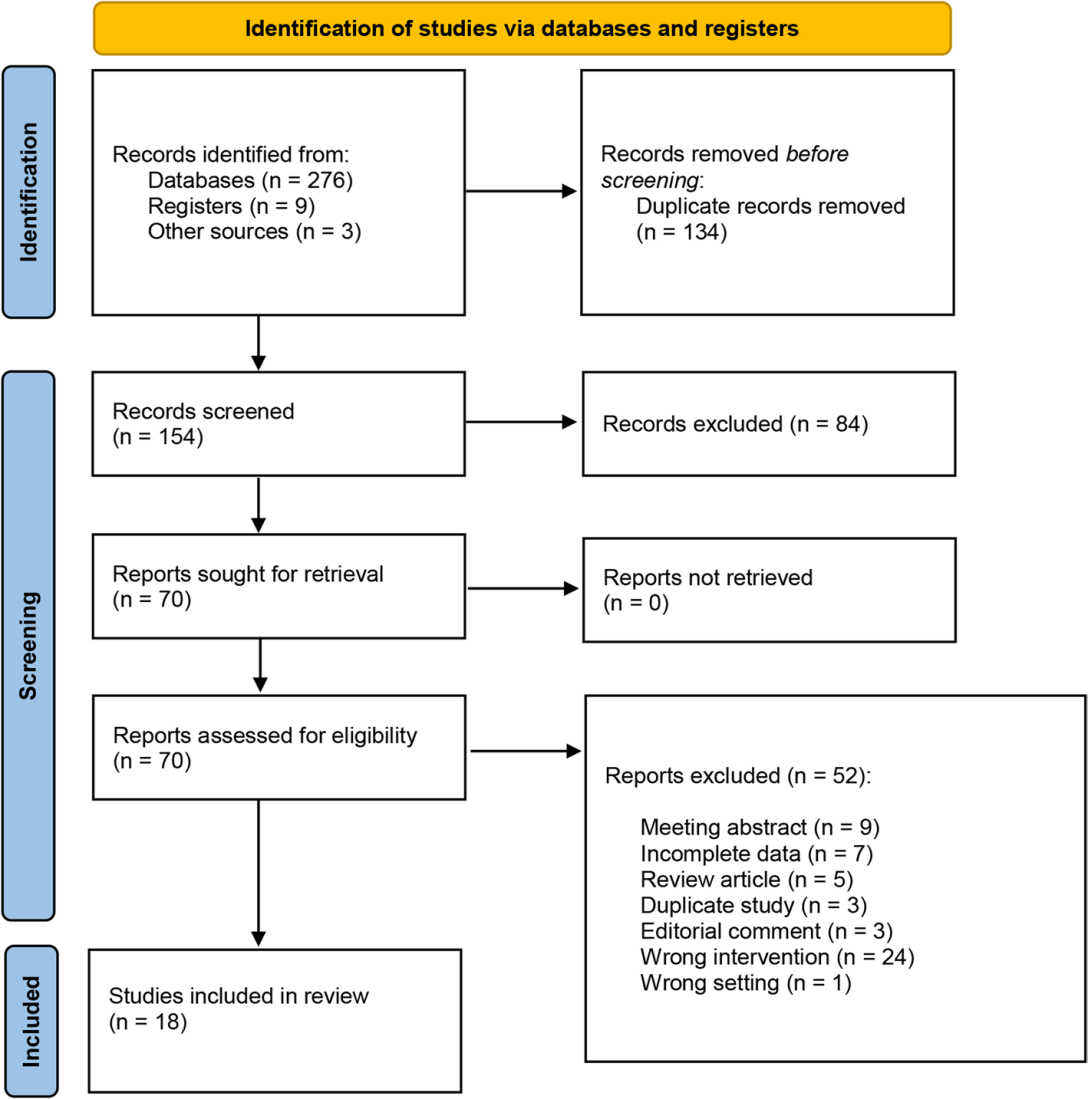
Flowchart of literature selection

The flow chart for the selection and final inclusion of the literature is shown in Fig. 1. After a thorough evaluation and elimination process, we finally found eighteen research studies [4–6, 15–29] out of a total of 288 research literature involving 3022 patients (1048 LRN vs. 1974 ORN). Four of these 18 controlled studies [6, 20, 22, 24] were prospective comparisons and five studies [6, 16, 19, 24, 26] were analyzed with matching. Five articles were included in cohort studies [6, 20, 22, 24, 26]. The baseline and oncologic characteristics of all included study patients are shown in Table 1. In addition, Table S2 provides a summary of the pathological parameters.

**Table 1:** Overview of collected studies

First author, year	Country	Design	Surgical method	No. of patients	Comparability	Tumor mean size (cm)	No. of IVC thrombus	Outcomes variables	Mean follow-up (months)	Study quality (score)
Lee et al. (2018) [25]	Korea	Retrospective	ORN/LRN	578/257	1, 2, 3, 5, 6, 7	9/8	0	1, 2, 3, 11	51.2/51.2	8
Nian et al. (2022) [26]	China	Retrospective	ORN/LRN	37/37	1, 2, 3, 4, 5, 7	11.7/11.3	0	1, 2, 3, 5, 6, 7, 8, 9, 10, 11	43/45.7	8
Hemal et al. (2007) [20]	India	Prospective	ORN/LRN	71/41	1, 2, 3, 4	10.1/9.9	NA	1, 4, 5, 6, 8, 9, 10, 11	57.2/51.4	9
Hattori et al. (2009) [4]	Japan	Retrospective	ORN/LRN	79/52	1, 2, 4, 6, 7	8.9/8.8	0	4, 5, 6, 8, 9, 11	51/41	7
Jeon et al. (2011) [5]	Korea	Retrospective	ORN/LRN	167/88	1, 2, 3, 4, 5, 7	9.8/9.2	0	1, 3, 4, 5, 6, 8, 9, 11	25.8/19	7
Steinberg et al. (2004) [27]	US	Retrospective	ORN/LRN	34/65	1, 2, 3, 4, 5, 6	9.9/9.2	0	6, 7, 9, 10, 11	NA	7
Chiba et al. (2016) [17]	Japan	Retrospective	ORN/LRN	42/37	1, 2, 3, 4	9.4/8.6	NA	4, 6, 7, 9, 10, 11	NA	7
Fang et al. (2013) [18]	China	Retrospective	ORN/LRN	317/71	1, 2, 3, 4, 6	9.5/8.8	0	6, 7, 9, 10, 11	NA	7
Huang et al. (2015) [19]	China	Retrospective	ORN/LRN	31/15	1, 2, 4, 5, 6, 7	13.2/12.1	0	1, 3, 5, 6, 8, 9, 10, 11	43.2/45.6	8
Zhu et al. (2016) [28]	China	Retrospective	ORN/LRN	68/84	1, 2, 3, 4, 6, 7	10.3/8.8	NA	4, 5, 6, 8, 9, 11	58.2/56.3	9
Kwon et al. (2011) [23]	Korea	Retrospective	ORN/LRN	35/33	1, 2, 3, 4, 7	9/8.2	NA	2, 4, 6, 9, 11	65.6/60	8
Fujita et al. (2014) [29]	Japan	Retrospective	ORN/LRN	124/83	1, 2, 4, 6, 7	9.3/8.7	NA	2, 3, 4, 5, 6, 8, 9, 11	109/71	6
Zhen et al. (2010) [21]	China	Retrospective	ORN/LRN	36/36	1, 2, 4	8.8/8.5	NA	5, 6, 7, 8, 9, 10, 11	15/15	6
Khan et al. (2019) [22]	India	Prospective	ORN/LRN	30/30	1, 2, 4, 7	NA	0	5, 6, 7, 8, 9, 10, 11	17.5/17	8
Laird et al. (2015) [24]	UK	Prospective	ORN/LRN	25/25	1, 2, 4, 6, 7	10/8.7	NA	1, 2, 3, 6, 7, 9, 10, 11	54/56.4	8
Dillenburg et al. (2006) [6]	Germany	Prospective	ORN/LRN	25/23	1, 2, 3, 4, 6, 7	9.3/8.9	0	5, 6, 7, 8, 9, 10, 11	13/12	9
Bensalah et al. (2009) [16]	France	Retrospective	ORN/LRN	135/44	1, 2, 7	NA	0	2	55/28	8
Bayrak et al. (2014) [15]	Turkey	Retrospective	ORN/LRN	140/33	1, 5, 6	9.9/9.5	0	5, 7, 8, 10	33/23	8

Comparability: 1 = age, 2 = gender, 3 = BMI, 4 = tumor side, 5 = ASA≥3, 6 = pathological stage ≥pT3, 7 = Fuhrman grade≥3

Outcomes variables:1 = OS, 2 = CSS, 3 = PFS, 4 = RFS, 5 = local recurrence, 6 = OT, 7 = LOS, 8 = EBL, 9 = blood transfusion 10 = intraoperative complications, 11 = postoperative complications

Study quality: The score of each study was allocated from 0 to 9 according to the modified Newcastle–Ottawa Scale and showed in Study quality

*LRN* Laparoscopic radical nephrectomy, *ORN* Open radical nephrectomy, *IVC* Inferior vena cava, *NA* Not available

### Assessment of quality

The scope of the research covered the period from 2004 to 2022, and the ROBINS-I tool showed that the overall bias of the study was moderate or higher (see Table S3 for details). Furthermore, we found that all included studies were moderate or better quality, as measured by the NOS score (> 5). Tables S4 and S5 present concrete evidence regarding the quality assessment.

### Baseline characteristic variable

No significant differences were observed between the ORN and LRN groups in terms of age (*p* = 0.44), gender (*p* = 0.11), BMI (*p* = 0.1), ASA ≥ 3 (*p* = 0.42), pT ≥ 3 (*p* = 0.09), and tumor laterality (*p* = 0.51). However, the proportion of people with Fuhrman grade ≥ 3 in the LRN group was more significant than in the ORN group (*p* = 0.02). Another noteworthy finding was that tumors in the ORN group tended to be larger on average than those in the LRN group, with a statistically significant difference (*p* < 0.00001) (Table 2).

**Table 2:** Baseline characteristic variable

Baseline characteristic	No. of studies	LRN vs. ORN	Heterogeneity *I*^2^ (%)	*p*-value
Age WMD (95% CI)	16	0.29 (−0.44, 1.01)	19	0.44
Male OR (95% CI)	17	1.14 (0.97, 1.35)	0	0.11
BMI WMD (95% CI)	9	0.16 (−0.03, 0.35)	0	0.1
Right side OR (95% CI)	15	1.07 (0.88, 1.30)	0	0.51
ASA≥3 WMD (95% CI)	5	0.82 (0.51, 1.33)	61	0.42
≥pT3 OR (95% CI)	10	0.75 (0.54, 1.04)	49	0.09
Fuhrman grade≥3 OR (95% CI)	12	1.27 (1.04, 1.56)	0	0.02
Tumor size WMD (95% CI)	16	0.64 (0.38, 0.9)	68	<0.00001

*LRN* Laparoscopic radical nephrectomy, *ORN* Open radical nephrectomy, *BMI* Body mass index, *ASA* American Society of Anesthesiologists score, *OR* Odds ratio, *WMD* Weighted mean difference, *CI* Confidence interval

### Survival outcome

#### Overall survival (OS)

**Fig. 2:**
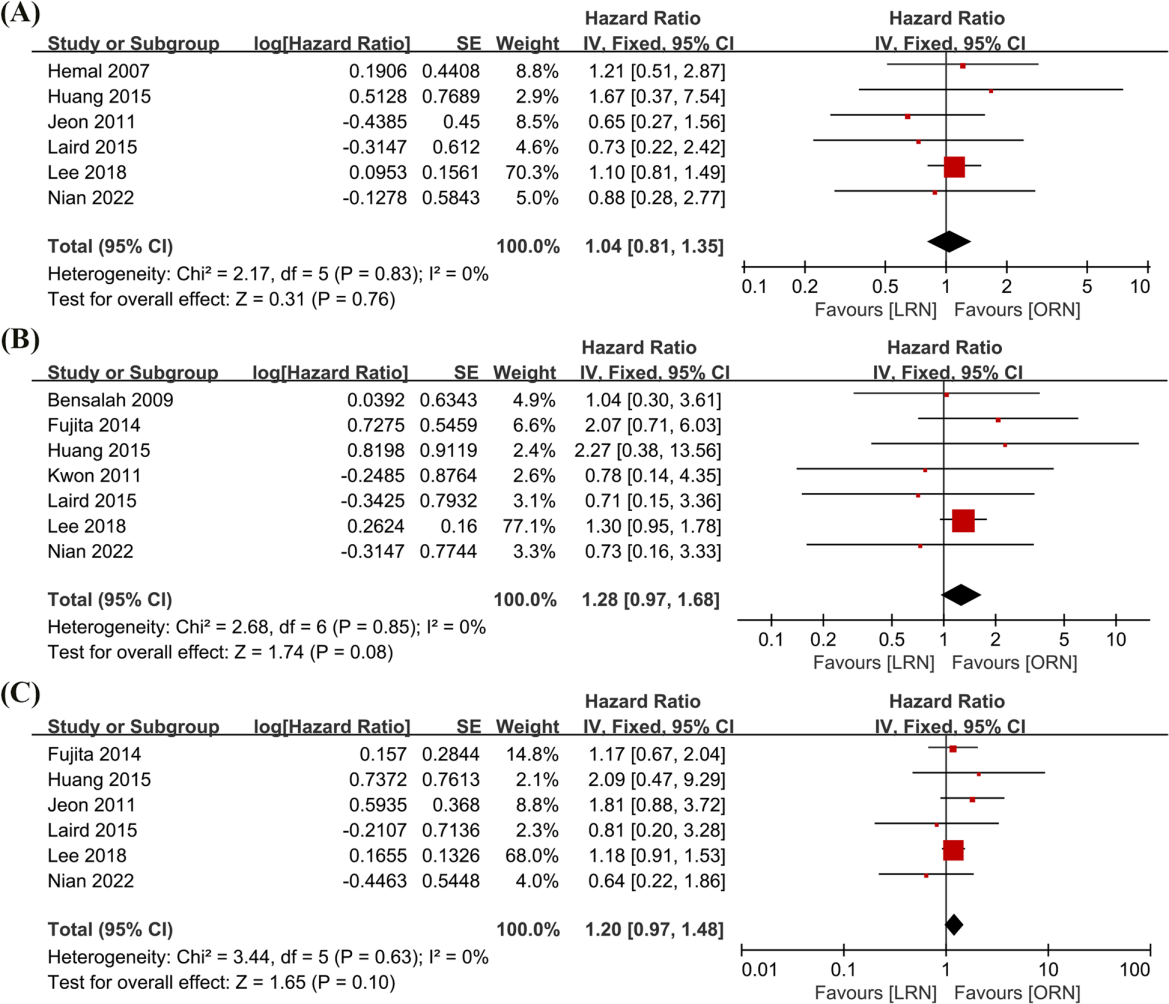
Forest plot of oncological outcomes: **A** Overall survival (OS). **B** Cancer-specific survival (CSS). **C** Progression-free survival (PFS)

A total of six studies [5, 12, 19, 20, 24–26] compared overall survival between LRN and ORN (463 cases in LRN and 909 cases in ORN). No significant heterogeneity was observed in the pooled studies (*I*^2^ = 0%, *p* = 0.83). Therefore, a fixed effects model was used to calculate the combined analysis. The meta-analysis revealed no statistically significant changes in OS between LRN and ORN during the follow-up period (HR = 1.04, 95% CI: 0.81 to 1.35, *p* = 0.76, Fig. 2A).

#### Cancer-specific survival (CSS)

Seven studies [16, 19, 23–26, 29] were included to compare cancer-specific survival between LRN and ORN (494 cases in LRN and 965 cases in ORN). For the results of the studies without heterogeneity, the fixed effects model was chosen to calculate and combine HR (*I*^2^ = 0%, *p* = 0.85). The findings revealed no discernible difference in CSS between the LRN and ORN groups (HR = 1.28, 95% CI: 0.97 to 1.68, *p* = 0.08, Fig. 2B).

#### Progression-free survival (PFS)

Six studies [5, 19, 24–26, 29] compared progression-free survival between LRN and ORN (505 cases in LRN and 962 cases in ORN). The results of the study showed no heterogeneity, and a fixed effect model was used for pooled analysis (*I*^2^ = 0%, *p* = 0.63). The combined results showed no significant difference in PFS between LRN and ORN (HR = 1.20, 95% CI: 0.97 to 1.48, *p* = 0.1, Fig. 2C).

#### Recurrence-free survival (RFS)

**Fig. 3:**
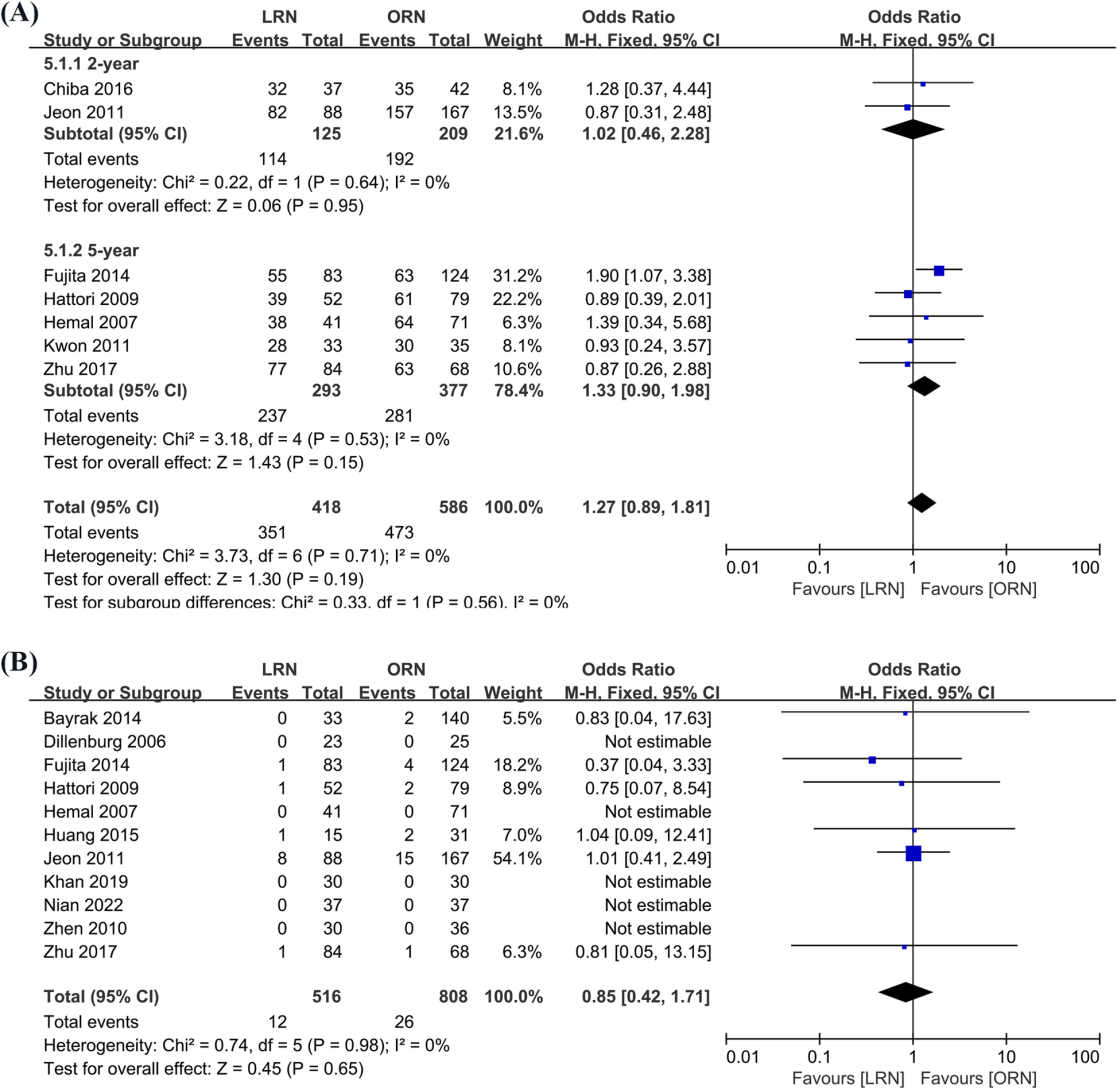
Forest plot of oncological outcomes: **A** Recurrence-free survival (RFS). **B** Local recurrence

Seven studies examined postoperative results for recurrence-free survival (418 cases in LRN and 516 cases in ORN), with two studies [5, 17] examining results for 2 years and five studies [4, 20, 23, 28, 29] observing results for 5 years. Based on the findings of the test for heterogeneity (*I*^2^ = 0%, *p* = 0.71), the corresponding studies were pooled and analyzed using the fixed effects model. At 2 or 5 years after surgery, we did not discover a statistically significant difference in RFS between LRN and ORN (OR = 1.27, 95% CI: 0.89 to 1.81, *p* = 0.56, Fig. 3A).

#### Local recurrence

According to a meta-analysis of the 10 included studies (479 cases in LRN and 771 cases in ORN) that used a fixed effects model (*I*^2^ = 0%, *p* = 0.98). LRN and ORN were not statistically significantly different and had similar local recurrence rates (OR = 0.85, 95% CI: 0.42 to 1.71, *p* = 0.65, Fig. 3B).

### Perioperative effectiveness

#### Operative time (OT) (min)

**Fig. 4:**
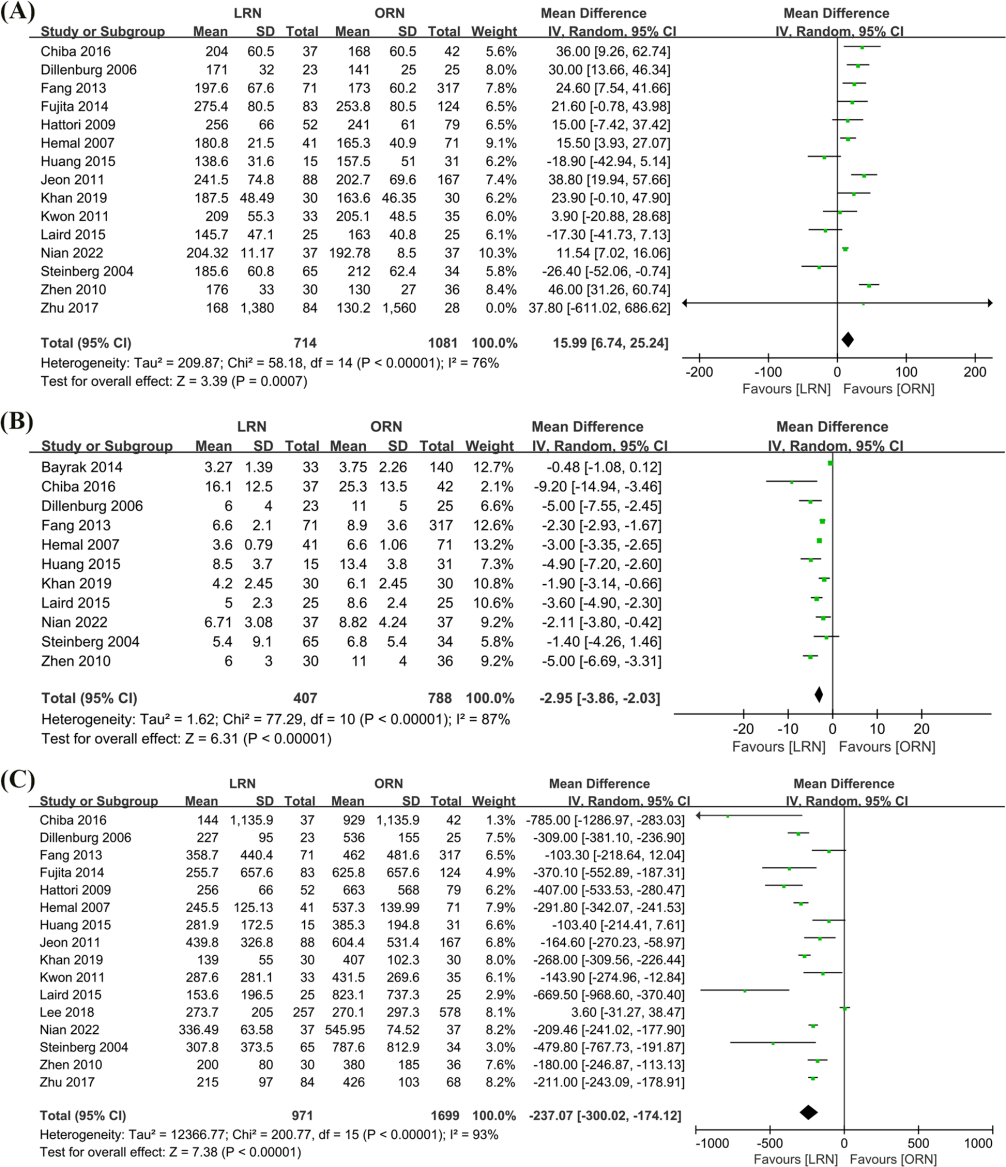
Forest plot of perioperative outcomes: **A** Operative time (min). **B** Length of stay (day). **C** Estimated blood loss (ml)

According to a meta-analysis of the 15 included studies (714 cases in LRN and 1081 cases in ORN) that used a random effects model with a high degree of heterogeneity (*I*^2^ = 76%, *p* < 0.00001). The operating times of the LRN group were longer and statistically significant compared to the ORN group (WMD = 15.99min, 95% CI: 6.74 to 25.24, *p* = 0.0007, Fig. 4A).

#### Length of stay (LOS) (day)

Based on a meta-analysis of the eleven included studies (407 cases in LRN and 788 cases in ORN) that used a random-effects model with a high degree of heterogeneity (*I*^2^ = 87%, *p* < 0.00001). The length of stay (LOS) was shorter and statistically significant in the LRN group compared to the ORN group (WMD = −2.95 days, 95% CI: −3.86 to −2.03, *p* < 0.00001, Fig. 4B).

#### Estimated blood loss (EBL) (ml)

A meta-analysis of 16 trials (971 cases in LRN and 1699 cases in ORN), combined using a random effects model with a high degree of heterogeneity (*I*^2^ = 93%, *p* < 0.00001), revealed that estimated blood loss (EBL) was statistically significantly lower in the LRN group than in the ORN group (WMD = −237.07ml, 95% CI: −300.02 to −174.12, *p* < 0.00001, Fig. 4C).

#### Blood transfusion rate

**Fig. 5:**
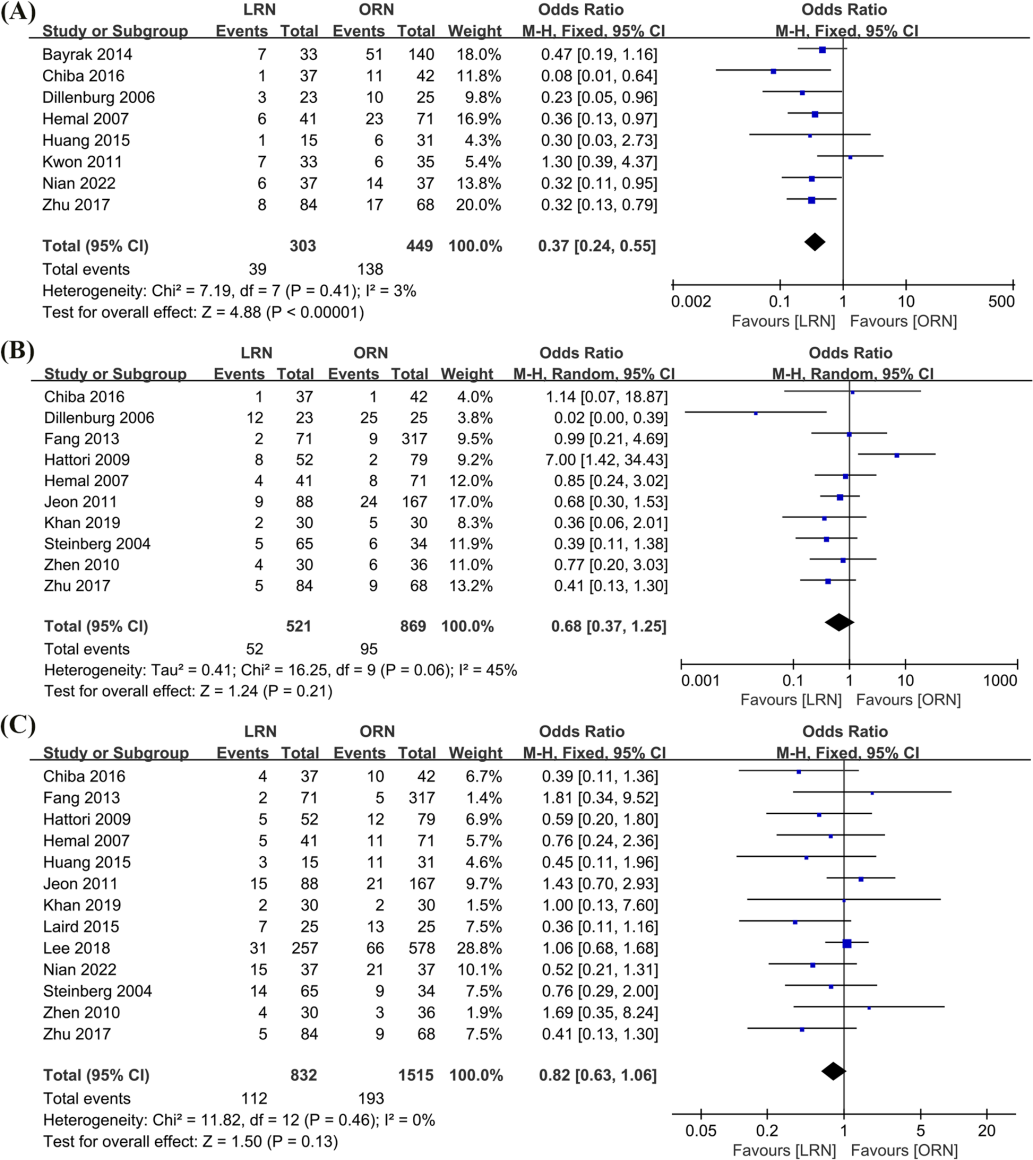
Forest plot of perioperative outcomes: **A** Blood transfusion rate. **B** Intraoperative complications. **C** Postoperative complications

A meta-analysis of eight included studies (303 cases in LRN and 449 cases in ORN), coupled using a fixed effects model (*I*^2^ = 3%, p = 0.41), revealed statistically significant differences in transfusion rates between the LRN and ORN groups (OR = 0.37, 95% CI: 0.24 to 0.55, *p* < 0.00001, Fig. 5A).

### Complications

#### Intraoperative complications

According to a meta-analysis of ten trials (521 cases in LRN and 861 cases in ORN) combined using a random-effects model (*I*^2^ = 45%, *p* = 0.06). The LRN group had significantly fewer intraoperative complications than the ORN group (OR = 0.68, 95% CI: 0.37 to 1.25, *p* = 0.21, Fig. 5B).

#### Postoperative complications

A meta-analysis of thirteen included studies (832 cases in LRN and 1515 cases in ORN), coupled using a fixed effects model (*I*^2^ = 0%, p = 0.46), revealed no significant differences between the LRN and ORN groups in terms of postoperative problems (OR = 0.82, 95% CI: 0.63 to 1.06, *p* = 0.13, Fig. 5C).

### Heterogeneity

Most studies revealed low to moderate heterogeneity, yet some outcomes such as OT, LOS, and EBL unavoidably presented high heterogeneity (*I*^2^>60%). Despite the tendency of surgeons to opt for open surgery when confronted with larger renal tumors, the presence of a time span of studies, and the bias of small samples, these factors should not be overlooked when reviewing the results.

### Analysis of sensitivity and publication bias

It is evident from the NOS score that most studies are of medium or higher quality. However, we can use sensitivity testing to confirm the validity of a few studies (OT, LOS, EBL) with substantial heterogeneity. After removing each trial individually and recalculating the aggregate mean difference, we discovered stable changes in the results (Figure S1). According to the funnel graph and the Egger regression tests (*p* > 0.05), the correlation meta-analysis for OT, LOS, and EBL did not reveal any evidence of publication bias (Figure S2).

## Discussion

This is the first evidence-based study to compare the short- and long-term prognostic outcomes following LRN and ORN surgery for large renal carcinomas (> 7cm). It is imperative that we must delve deeper into the key findings of the investigation. In the treatment of giant renal carcinoma, the findings of this meta-analysis did not reveal significant differences between LRN and ORN in the prognostic outcome indicators OS, CSS, PFS, RFS, local recurrence, and complications, while the partial perioperative outcomes OT, LOS, EBL, and transfusion rate were comparable and statistically different.

### Prognostic outcome

The research showed older males, low BMI, and large tumor size were significantly poor prognostic factors for PFS, OS, and CSS, while the type of surgery was not significantly associated with postoperative survival outcome [25]. Lou et al. [30] studied the oncologic outcomes of limited renal cell carcinoma (pT1-pT2) with a median follow-up of approximately 4 years and found that LRN and ORN had similar outcomes, while tumor stage was an independent prognostic factor for CSS of localized RCC. It is expected that stage pT3 tumors have a lower 5-year CSS of 67% and OS of 62%, considering the correlation between larger tumor size and poorer survival [31], which is closely related to tumor complexity and operator proficiency.

Analysis of the enrolled patients revealed that lymph node enlargement was not commonly found, and it is currently not recommended to perform lymph node dissection (LND) routinely during radical nephrectomy for RCC. However, retrospective studies [32] have suggested that concomitant LND may be beneficial for high-risk patients with preoperative or intraoperative regional lymph node enlargement. Capitanio et al. [33] found that the number of lymph nodes removed by LND was associated with disease-free survival (DFS) and CSS in patients with tumor volume >10 cm and pathological stages of pT2/pT3c/pT4.

Due to the rigidity of laparoscopic instruments, limited range of motion, and difficulties with suturing, traditional open surgery is generally adopted for patients with RCC who have concomitant inferior vena cava (IVC) thrombus, which inevitably brings certain bias to this study. Although the cumulative analysis showed that LRN and ORN had similar oncologic outcomes, we cannot exclude a partial bias in outcome by the duration of follow-up.

### Surgical outcomes

The prolonged operative time for LRN versus ORN could be partly attributed to the learning curve and the lack of standardization in intraoperative protocols [20]. Nevertheless, no negative patient outcomes were observed and it may not be economically beneficial, as it led to shorter hospitalization and recovery time. The estimated intraoperative blood loss and blood transfusion would increase with increasing cancer volume. Early posterior laparoscopic ligation of renal tubular vessels reduces bleeding during intraoperative dissection [34]. The surgeon can perform more precise dissection and hemostasis under minimally invasive surgery with clear vision and complete instrumentation because larger renal carcinomas have more parasitic vessels surrounding them. Gu et al. [35] showed that robot-assisted radical nephrectomy (RARN) provided better control of EBL compared to ORN in the treatment of renal carcinoma combined with IVC thrombosis (250 vs. 1000 ml, *p* < 0.001).

### Complications

In general, laparoscopic surgery is associated with fewer complications than open surgery. Dillenburg et al. [6] showed that retroperitoneal laparoscopic radical nephrectomy (RLRN) was more advantageous than ORN in intraoperative complications (52% vs. 100%, *p* < 0.001) and with similar short-term oncologic outcomes. The retroperitoneal approach simplifies the surgical procedure, providing a direct route to the kidney, but is limited by the narrow space and poor maneuverability of laparoscopic instruments. The advantage of the transperitoneal laparoscopic radical nephrectomy (TLRN) is a wider working space and more easily recognizable anatomical landmarks; however, there is a risk of adhesion lysis and bowel mobilization. A previous meta-analysis showed that RLRN had lower overall complications compared to TLRN (*p* = 0.03) [36]. Although no significant difference was found in the complication rate between LRN and ORN in large-volume renal tumors, it is important to consider the interference of patient heterogeneity, as the tumor volume was larger in the ORN group. Therefore, caution should be taken when interpreting the results.

The use of robot-assisted radical nephrectomy (RARN) for renal cell carcinoma remains controversial. The latest meta-analysis did not exhibit significant differences in patient perioperative outcomes between the two surgical approaches [37, 38]. Furthermore, although a shorter hospital stay may reduce overall cost, the pooled analysis shows that RARN is more expensive than LRN [38, 39]. RARN mainly deals with patients with local infiltration and combined venous thrombosis, and RARN seems to offer greater flexibility for resection and suturing [40]. Both RARN and LRN showed more satisfactory results than ORN. More prospective studies are needed in the future to discuss the differences between RARN and LRN in the treatment of large-volume renal cell carcinoma.

### Limitation

Our research has some limitations. Firstly, there is a lack of randomized controlled studies in our study, as most of the controlled observational studies used databases with potential bias and misclassification. Furthermore, there was a lack of comparative results based on tumor stage. Secondly, the study did not specify whether the patients were combined with drugs for follow-up. Some studies had small sample sizes and shorter follow-up periods, lacking long-term oncologic results. Thirdly, although some evidence was presented by sensitivity analysis for some more heterogeneous results, confounding factors were inevitable and should be interpreted with caution. Finally, there may be unknown differences between geographical regions and medical institutions, making generalizing reported results difficult.

## Conclusion

In conclusion, the result of our meta-analysis recommends that even if the OT for LRN is longer, LRN offers specific perioperative advantages (LOS, EBL, and transfusion rates) over ORN for patients undergoing radical nephrectomy for tumor volume (> 7 cm). Despite this, there are no differences in complications or oncological outcomes. The superiority of LRN over ORN will need to be proven in future large prospective randomized controlled trials.

## Supplementary Information

**Additional file 1: Table S1.** PRISMA 2020 Checklist. **Table S2.** Pathological parameters. **Table S3.** Risk of bias evaluation of non-randomized studies using the ROBINS-I tool. **Table S4.** Study quality of case–control studies based on the Newcastle-Ottawa scale. **Table S5.** Study quality of cohort studies based on the Newcastle-Ottawa scale.

**Additional file 2: Figure S1.** Sensitivity analysis of perioperative outcomes: (A) Operative time (min); (B) Length of stay (day); (C) Estimated blood loss (ml).

**Additional file 3: Figure S2.** Forest plot to explore publication bias: (A) Operative time (min); (B) Length of stay (day); (C) Estimated blood loss (ml).

## Data Availability

The article and the supplementary material contain all datasets produced by this investigation.

## Abbreviations

LRN: Laparoscopic radical nephrectomy
ORN: Open radical nephrectomy
RCC: Renal cell carcinoma
WMD: Weighted mean difference
HR: Hazard ratio
OR: Odds ratio
Cl: Confidence intervals
OS: Overall survival
CSS: Cancer-specific survival
RFS: Recurrence-free survival
PFS: Progression-free survival
DFS: Disease-free survival
OT: Operative time
EBL: Estimated blood loss
LOS: Length of stay
PN: Partial nephrectomy
BMI: Body mass index
ASA: American Society of Anesthesiologists
NOS: Newcastle-Ottawa Scale
SD: Standard deviation
IQR: Interquartile range
TLRN: Transperitoneal laparoscopic radical nephrectomy
RLRN: Retroperitoneal laparoscopic radical nephrectomy
RARN: Robot-assisted radical nephrectomy
IVC: Inferior vena cava
LND: Lymph node dissection

## References

[1] Sung H, Ferlay J, Siegel RL, Laversanne M, Soerjomataram I, Jemal A (2021). Global cancer statistics 2020: GLOBOCAN estimates of incidence and mortality worldwide for 36 cancers in 185 countries. CA Cancer J Clin.

[2] Sun M, Thuret R, Abdollah F, Lughezzani G, Schmitges J, Tian Z (2011). Age-adjusted incidence, mortality, and survival rates of stage-specific renal cell carcinoma in North America: a trend analysis. Eur Urol.

[3] Clayman RV, Kavoussi LR, Soper NJ, Dierks SM, Meretyk S, Darcy MD (1991). Laparoscopic nephrectomy: initial case report. J Urol.

[4] Hattori R, Osamu K, Yoshino Y, Tsuchiya F, Fujita T, Yamada S (2009). Laparoscopic radical nephrectomy for large renal-cell carcinomas. J Endourol.

[5] Jeon SH, Kwon TG, Rha KH, Sung GT, Lee W, Lim JS (2011). Comparison of laparoscopic versus open radical nephrectomy for large renal tumors: a retrospective analysis of multi-center results. BJU Int.

[6] Dillenburg W, Poulakis V, Skriapas K, de Vries R, Ferakis N, Witzsch U (2006). Retroperitoneoscopic versus open surgical radical nephrectomy for large renal cell carcinoma in clinical stage cT2 or cT3a: quality of life, pain and reconvalescence. Eur Urol.

[7] Wells GA, Shea B, O’Connell D, Peterson J, Welch V, Losos M, et al. The Newcastle-Ottawa Scale (NOS) for assessing the quality of nonrandomised studies in meta-analyses. Oxford; 2000.

[8] Stang A (2010). Critical evaluation of the Newcastle-Ottawa scale for the assessment of the quality of nonrandomized studies in meta-analyses. Eur J Epidemiol..

[9] Sterne JA, Hernán MA, Reeves BC, Savović J, Berkman ND, Viswanathan M (2016). ROBINS-I: a tool for assessing risk of bias in non-randomised studies of interventions. BMJ (Clinical research ed)..

[10] Luo D, Wan X, Liu J, Tong T (2018). Optimally estimating the sample mean from the sample size, median, mid-range, and/or mid-quartile range. Stat Methods Med Res.

[11] McGrath S, Zhao X, Steele R, Thombs BD, Benedetti A (2020). Estimating the sample mean and standard deviation from commonly reported quantiles in meta-analysis. Stat Methods Med Res.

[12] Tierney JF, Stewart LA, Ghersi D, Burdett S, Sydes MR (2007). Practical methods for incorporating summary time-to-event data into meta-analysis. Trials..

[13] Sterne JA, Gavaghan D, Egger M (2000). Publication and related bias in meta-analysis: power of statistical tests and prevalence in the literature. J Clin Epidemiol.

[14] Lau J, Ioannidis JP, Terrin N, Schmid CH, Olkin I (2006). The case of the misleading funnel plot. BMJ (Clinical research ed)..

[15] Bayrak O, Seckiner I, Erturhan S, Cil G, Erbagci A, Yagci F (2014). Comparison of the complications and the cost of open and laparoscopic radical nephrectomy in renal tumors larger than 7 centimeters. Urol J.

[16] Bensalah K, Salomon L, Lang H, Zini L, Jacqmin D, Manunta A (2009). Survival of patients with nonmetastatic pT3 renal tumors: a matched comparison of laparoscopic vs open radical nephrectomy. BJU Int.

[17] Chiba K, Kamada S, Yamamoto S, Okato A, Inoue T, Nozumi K (2016). L aparoscopic radical nephrectomy for large renal cell carcinoma: retrospective analysis of safety and oncological outcome. Nihon Hinyokika Gakkai Zasshi.

[18] Fang D, Yang K, Li X, Yang X, Tang Q, Tang Y (2013). Comparison of clinical outcomes of laparoscopic versus open radical nephrectomy for large renal tumors. J Modern Urol.

[19] Huang H, Huang Y, Pan X, Li L, Chen J, Yin L (2015). Matched-pair study on laparoscopic versus open radical nephrectomy for the treatment of large renal tumor. J Clin Urol.

[20] Hemal AK, Kumar A, Kumar R, Wadhwa P, Seth A, Gupta NP (2007). Laparoscopic versus open radical nephrectomy for large renal tumors: a long-term prospective comparison. J Urol.

[21] Zhen J, Yan Y, Peng B, Chao Y, Xu Y, Zhang H (2010). Laparoscopic radical nephrectomy for clinical stage T2 renal cell carcinoma patients. Chinese J Urol.

[22] Khan MMA, Patel RA, Jain N, Balakrishnan A, Venkataraman M (2019). Prospective analysis of laparoscopic versus open radical nephrectomy for renal tumors more than 7 cm. J Minimal Access Surg..

[23] Kwon SY, Jung JW, Kim BS, Kim TH, Yoo ES, Kwon TG (2011). Laparoscopic versus open radical nephrectomy in T2 renal cell carcinoma: long-term oncologic outcomes. Korean J Urol.

[24] Laird A, Choy KC, Delaney H, Cutress ML, O'Connor KM, Tolley DA (2015). Matched pair analysis of laparoscopic versus open radical nephrectomy for the treatment of T3 renal cell carcinoma. World J Urol.

[25] Lee H, Lee CU, Yoo JH, Sung HH, Jeong BC, Jeon SS (2018). Comparisons of oncological outcomes and perioperative complications between laparoscopic and open radical nephrectomies in patients with clinical T2 renal cell carcinoma (≥7cm). PloS one..

[26] Nian X, Ye H, Zhang W, Zhang K, Gao X, Yang B (2022). Propensity-matched pair analysis of safety and efficacy between laparoscopic and open radical nephrectomy for the treatment of large renal masses (>10 cm): a retrospective cohort study. Transl Androl Urol.

[27] Steinberg AP, Finelli A, Desai MM, Abreu SC, Ramani AP, Spaliviero M (2004). Laparoscopic radical nephrectomy for large (greater than 7 cm, T2) renal tumors. J Urol.

[28] Zhu X, Yang X, Hu X, Zhang X (2016). Retroperitoneoscopic versus open surgical radical nephrectomy for 152 Chinese patients with large renal cell carcinoma in clinical stage cT2 or cT3a: A long-term retrospective comparison. J Cancer Res Ther.

[29] Fujita T, Hattori R, Yoshino N, Kimura T, Kamidaira O, Yamada S (2014). Laparoscopic radical nephrectomy for large renal cell carcinoma(>7cm). Jpn J Endourol.

[30] Luo JH, Zhou FJ, Xie D, Zhang ZL, Liao B, Zhao HW (2010). Analysis of long-term survival in patients with localized renal cell carcinoma: laparoscopic versus open radical nephrectomy. World J Urol.

[31] Bhindi B, Thompson RH, Lohse CM, Mason RJ, Frank I, Costello BA (2018). The probability of aggressive versus indolent histology based on renal tumor size: implications for surveillance and treatment. Eur Urol.

[32] Blom JH, van Poppel H, Maréchal JM, Jacqmin D, Schröder FH, de Prijck L (2009). Radical nephrectomy with and without lymph-node dissection: final results of European Organization for Research and Treatment of Cancer (EORTC) randomized phase 3 trial 30881. Eur Urol.

[33] Capitanio U, Suardi N, Matloob R, Roscigno M, Abdollah F, Di Trapani E (2014). Extent of lymph node dissection at nephrectomy affects cancer-specific survival and metastatic progression in specific sub-categories of patients with renal cell carcinoma (RCC). BJU Int.

[34] Yang Q, Du J, Zhao ZH, Chen XS, Zhou L, Yao X (2013). Fast access and early ligation of the renal pedicle significantly facilitates retroperitoneal laparoscopic radical nephrectomy procedures: modified laparoscopic radical nephrectomy. World J Surg Oncol.

[35] Gu L, Ma X, Gao Y, Li H, Li X, Chen L (2017). Robotic versus open level I-II inferior vena cava thrombectomy: a matched group comparative analysis. J Urol.

[36] Fan X, Xu K, Lin T, Liu H, Yin Z, Dong W (2013). Comparison of transperitoneal and retroperitoneal laparoscopic nephrectomy for renal cell carcinoma: a systematic review and meta-analysis. BJU Int.

[37] Li J, Peng L, Cao D, Cheng B, Gou H, Li Y (2020). Comparison of perioperative outcomes of robot-assisted vs. laparoscopic radical nephrectomy: a systematic review and meta-analysis. Front Oncol.

[38] Crocerossa F, Carbonara U, Cantiello F, Marchioni M, Ditonno P, Mir MC (2021). Robot-assisted radical nephrectomy: a systematic review and meta-analysis of comparative studies. Eur Urol.

[39] Gershman B, Bukavina L, Chen Z, Konety B, Schumache F, Li L (2020). The association of robot-assisted versus pure laparoscopic radical nephrectomy with perioperative outcomes and hospital costs. Eur Urol Focus..

[40] Chapman TN, Sharma S, Zhang S, Wong MK, Kim HL (2008). Laparoscopic lymph node dissection in clinically node-negative patients undergoing laparoscopic nephrectomy for renal carcinoma. Urology..

