# Supplementary figures and images for: Oncologic and perioperative outcomes of laparoscopic versus open radical nephrectomy for the treatment of renal tumor (> 7 cm): a systematic review and pooled analysis of comparative outcomes
Source: World J Surg Oncol. 2023 Feb 6;21:35. doi: 10.1186/s12957-023-02916-y (PMC9901136; doi:10.1186/s12957-023-02916-y)

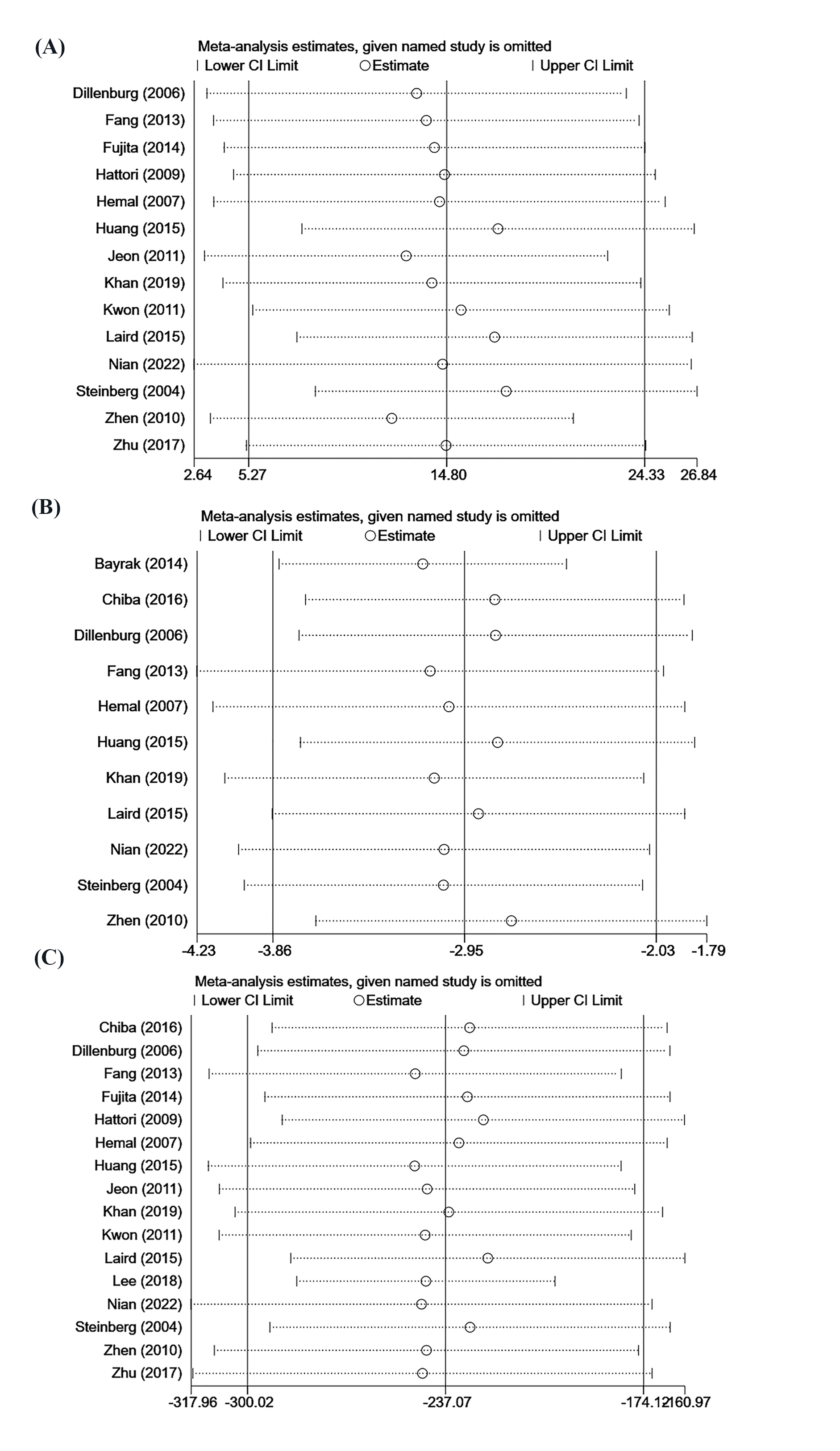

Supplement: Supplementary file 2 — Additional file 2: Figure S1. Sensitivity analysis of perioperative outcomes: (A) Operative time (min); (B) Length of stay (day); (C) Estimated blood loss (ml). [file 12957_2023_2916_MOESM2_ESM.tif]

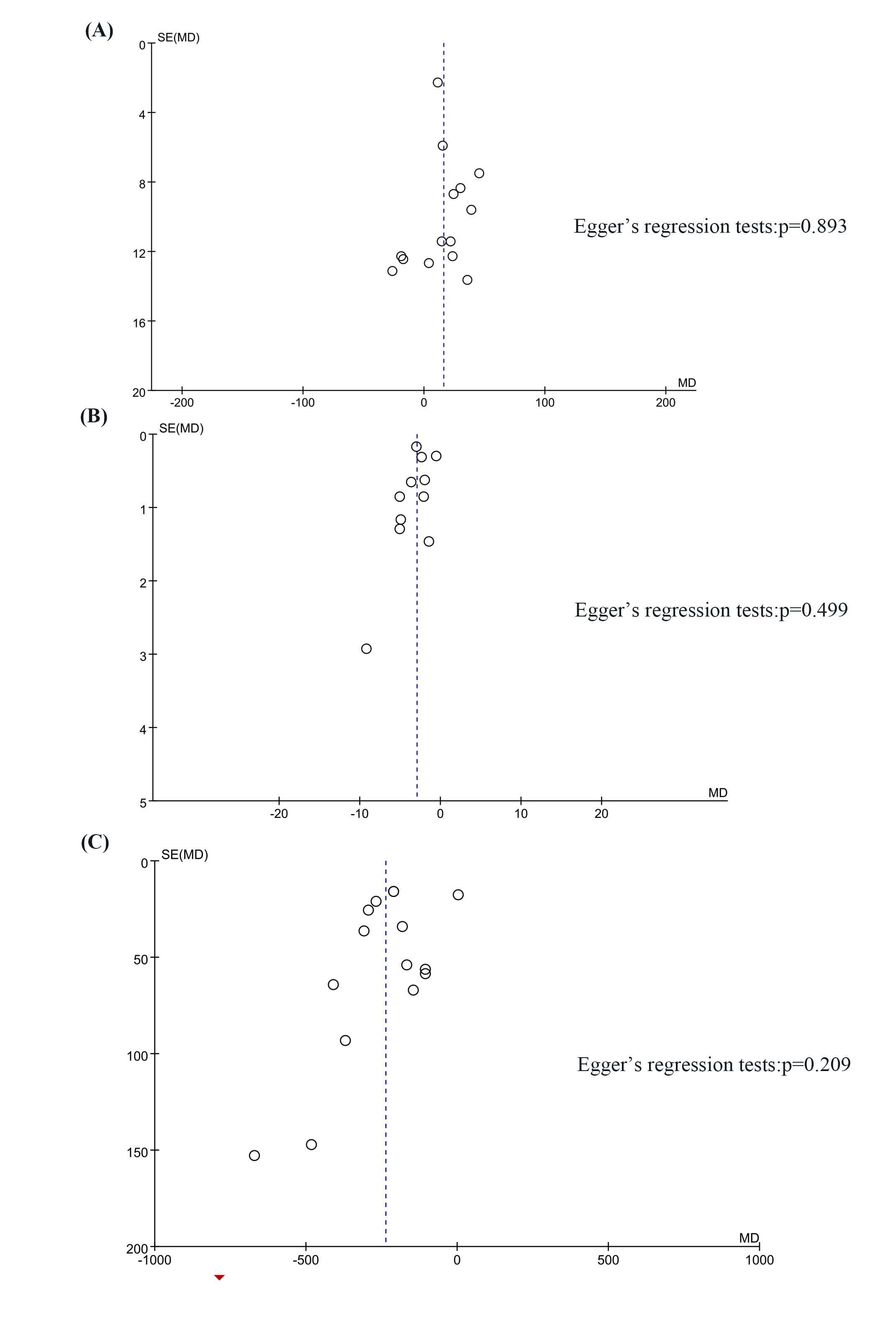

Supplement: Supplementary file 3 — Additional file 3: Figure S2. Forest plot to explore publication bias: (A) Operative time (min); (B) Length of stay (day); (C) Estimated blood loss (ml). [file 12957_2023_2916_MOESM3_ESM.tif]
